# Cough: impact, beliefs, and expectations from a national survey

**DOI:** 10.1186/s40248-016-0072-1

**Published:** 2016-09-27

**Authors:** Roberto W. Dal Negro, Massimiliano Mazzolini, Paola Turco, Alessandro Zanasi

**Affiliations:** 1 National Centre for Respiratory Pharmacoeconomics and Pharmacoepidemiology- CESFAR, Verona, Italy; 2 Department of Specialist-Diagnostic and Experimental Medicine (DIMES), Respiratory and Critical Care Medicine, S. Orsola-Malpighi Hospital, University of Bologna, Bologna, Italy; 3 Research & Clinical Governance, Verona, Italy; 4 Italian Association for Cough Study (AIST), Bologna, Italy

**Keywords:** CATI, Cough, Cough impact, National survey, People beliefs, Public opinion

## Abstract

**Background:**

Cough is one of the most common discomforts affecting general population, which can disrupt subjects’ quality of life due to its physical, social, and psychological effects. Aim of the study was to investigate the impact of cough and related beliefs of general population.

**Methods:**

A cross-sectional telephone survey was carried out by means of a specific, validated questionnaire on a representative sample of Italian general population. All the interviews were carried out according to the Computer Assisted Telephone Interview (CATI) methodology by expert, professional interviewers. Distributions of all answers were calculated in the overall sample.

**Results:**

A total of 1,251 subjects (mean age: 49 years; females 44.2 %) completed the interviews. The overall number of telephone calls was 5362, and the corresponding redemption rate was 1/4.3 (23.%). The sample was representative of national population in terms of geographical distribution, age, gender, and smoking habit. The majority of respondents was convinced that cough is merely a symptom of several different diseases, but 46.4 % of respondents affirmed that cough should be regarded as “a disease” per se. Only 29.1 % of subjects say that they usually do not complain of any cough over the year, while 18.4 % reported ≥ 3 episodes of cough/year. These episodes have a duration ranging 10–30 days in 19.9 % of subjects, and > 30 days in 6.9 % of subjects. The majority of respondents is worried about their cough only after 1 week, but 76.9 % of subjects is much more worried if cough affects a child. After a few days of cough, 23.1 % of subjects use domestic remedies; 20.9 % ask their pharmacist, and 33.4 % their doctor, being GPs (69.6 %) and lung physicians (16.2 %) the most asked professionals. The occurrence of bronchitis, pneumonia, upper airway infections, and allergic troubles are the most feared events. The majority of respondents are convinced that antibiotics and steroids should not be regarded as the gold standard for treating persistent cough (61.2 and 58.2 %, respectively), while anti-tussive drugs and aerosols in general are regarded as the most effective strategies (69.1 and 74.1 %, respectively). Moreover, 33.8 % of the sample is in favour of homeopathic drugs, while 23.2 % had already used an homeopathic anti-tussive syrup, and 27.6 % of subjects are really interested in using the homeopathic approach. The willingness to pay for an effective anti-tussive remedy was: 46.3 % up to 10 €; 27.8 % up to 20 €, and 13.3 % more than 20 €.

**Conclusions:**

Cough confirms its high impact in Italy, and a substantial proportion of individuals regards cough as “a disease”. Only one out of three Italians refers to their doctor, but when cough is already persistent. Cough in children is much more feared than in adults. The majority of Italians have a proper and conservative position versus both antibiotic and the systemic steroid uses against cough. The Italian attitude to aerosol therapy confirms very high. Differently from the cough guidelines, anti-tussive drugs are highly valued among Italian people. The attitude and the interest to homeopathic anti-tussive remedies proves high. Finally, the willingness to pay for an effective anti-tussive remedy is quite high in Italy.

**Electronic supplementary material:**

The online version of this article (doi:10.1186/s40248-016-0072-1) contains supplementary material, which is available to authorized users.

## Background

Cough is one of the most frequent events for which patients ask for a medical consult and it can represent a true challenge in daily practice.

There are epidemiological, patient-related, and therapeutic factors which may complicate the etiological diagnosis and the management of cough. Acute cough is frequently linked to common cold and upper respiratory tract infections (URTIs) [1]. Physical impairment is a frequent complication of cough, while more psychological aspects (such as, depression and social retirement) emerge when cough becomes chronic [2]. The impact of cough on a patient’s life ranges from minimal discomfort to disabling symptoms. Indeed, patients presenting acute cough may experience transient illness due to URTIs, which leads to lose work and school days, and increase the overall cost for cough management [3, 4]. On the other hand, patients often seek for medical attention due to their poor knowledge and unawareness of cough. Usually, subjects’ concerns tend to increase when their cough lasts for more than a week, in contrast with the expected duration of acute cough [3]. In real life, the net result is often the seek for a consult and of a pharmacological treatment, even if the most prescribed therapeutic options lack of a strong evidence of efficacy [5].

Aim of the present investigation was to carry out a national survey on cough impact and beliefs among adults individuals of general population.

## Methods

A cross-sectional telephone survey was carried out between July 21^st^-29^th^, 2015 on individuals aged ≥ 18 years. The principal instrument used by investigators to collect data was a specific, validated questionnaire. The questionnaire consisted of 24 simple pre-determined questions: 20/24 were closed questions (83.3 %), while question # 5; 6; 20, and 24 were open (Additional file 1). Possible answers for closed questions were “Yes”; “No”; “doubtful”. As concerning open questions # 5; # 6, and # 24, all respondents had to indicate their own opinion related to each specific question, while question # 20 attained to their age to be reported in years.

All interviews were carried out according to the Computer Assisted Telephone Interview (CATI) methodology [6] by expert, professional interviewers. Distributions of all answers were calculated in the overall sample. The interviewer was provided with one “work station” consisting of a personal computer connected to a central processing unit. The central unit was also equipped with a specific software for the random choice of individuals (such as, the telephone numbers) to contact. Compared to a conventional telephone interview, the CATI technique also allowed to randomize the questions to put. Moreover, this system worked as a supervisor of the interviewer’s activity: if the interviewer forgot some questions or even an entire section of the questionnaire, the pc would have alert him, thus avoiding errors due to missed questions. As previously mentioned, the sampling strategy adopted in the present survey was the random selection of an adequate number of subjects.

A minimum number of 1178 respondents was previously calculated for achieving the representativity of Italian population in terms of age, gender, education, smoking habit, and national geographical distribution (by 3 % maximum error, and 95 % probability) [6].

All interviews were always preceded by a short explanation of the aim of the survey, and had a mean duration of 5 min.

## Results

A total of 1,251 subjects (mean age: 49 years; females 44.2 %) completed the interview. The overall telephone contacts were 5362, and the corresponding redemption rate (such as the proportion of call properly completed and reliable for the investigation) was 1/4.3 (23.%).

The geographical distribution of respondents throughout Italy was: from Northern = 45.0 %; Central = 14.5, and Southern Regions = 42.9 %. Active smokers were 21.4 %, and ex-smokers 12.4 %.

As concerning the job distribution, white collars were 36.0 %, while retired persons were 24.8 %; housewives 14.6 %; blue collars 14.5 %, and students 9.7 %, respectively, while non-respondents to this question were 0.4 %.

Responses to the questionnaire’s questions were divided in five groups, according to their main intrinsic meaning, namely: 1) subjects’ basic convincement on cough; 2) cough general impact; 3) subjects’ approach to cough; 4) subjects’ therapeutic expectations, and 5) willingness to pay for managing cough effectively.

1. Subjects’ basic belief
  Even if the majority of respondents was convinced that cough has to be merely considered as a symptom of several different diseases, a substantial (46.4 %) proportion of respondents claimed that cough should be regarded as “a disease” per se (Fig. 1a and b).

**Fig. 1:**
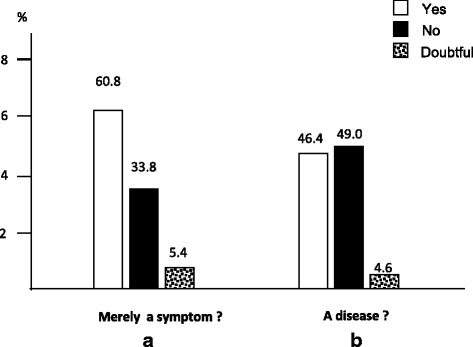
How is cough perceived among the general population. Beliefs of subjects who regarded cough merely as a symptom (panel **a**), rather than a disease (panel **b**)
2. General clinical impact
  Only 29.1 % of subjects reported that they usually never have cough over the year, while 18.4 % of them reported ≥ 3 episodes of cough/year (Fig. 2a). These episodes were described as having a different duration, ranging from 10 to 30 days in 19.9 % of subjects, and longer than 30 days in 6.9 % of subjects (Fig. 2b). Dry cough was described as slightly more frequently occurring (53.2 % vs 45.1 %), even if cough with secretions was more frequent in respondents aged < 18 years.

**Fig. 2:**
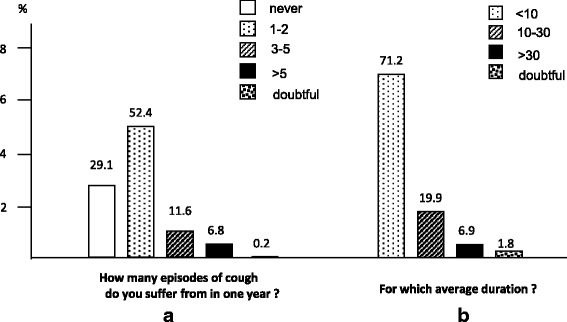
Mean annual frequency (panel **a**) and duration (panel **b**) of cough episodes
  The majority of respondents (71.7 %) worried about their own cough only after 1-week duration, even if the great majority of subjects (76.9 %) affirmed to be worried about cough much earlier and much more if cough affects a child.
3. Subjects’ approach to cough
  Moreover, while 21.0 % of respondents declared to remain still waiting during the first days of cough, 23.1 % of respondents affirmed that they start using domestic remedies for managing their cough quite early; 20.9 % ask their pharmacist for any pharmacological support, and 33.4 % call their GPs for a medical consultation after a few days of cough (Fig. 3a and b).

**Fig. 3:**
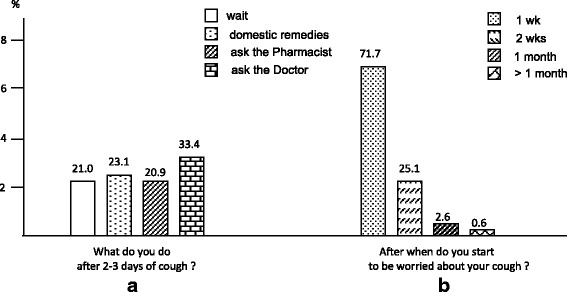
The general subjects’ approach to cough. What subjects do after 2-3 days of cough (panel **a**), and after when subjects start to be worried about their cough (panel **b**)
  GPs (69.6 %) and lung physicians (16.2 %) resulted the most asked professionals for cough. The occurrence of bronchitis, rather than pneumonia, or upper airway infections, or allergic troubles were the four most feared events.
4. Subjects’ therapeutic expectations
  The majority of respondents affirmed that antibiotics and steroids should not be regarded as the gold standard for treating persistent cough (61.2 and 58.2 %, respectively) (Fig. 4a and b), while anti-tussive drugs and aerosol therapy were generally regarded as the most effective therapeutic options (69.1 and 74.1 %, respectively) (Fig. 5a and b).

**Fig. 4:**
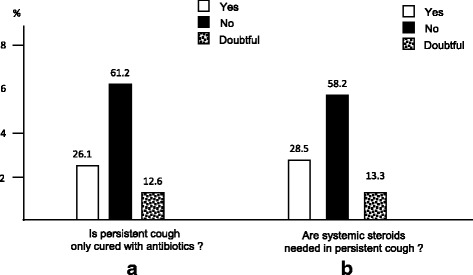
Patients’ opinion on antibiotics (panel **a**) and systemic steroids (panel **b**) use in persistent cough

**Fig. 5:**
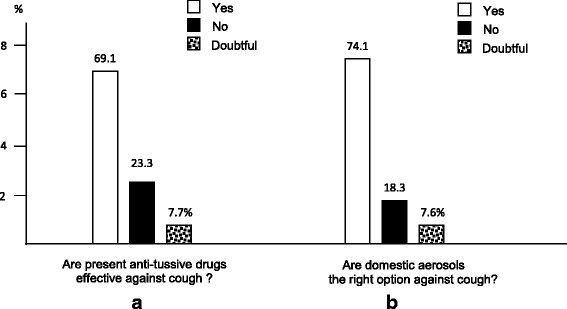
Patients’ opinion on present anti-tussive drugs (panel **a**) and aerosols (panel **b**) in persistent cough
  Moreover, 33.8 % of the sample declared to be in favour of homeopathic drugs (Fig. 6), while 23.2 % had already used an homeopathic anti-tussive syrup, and 27.6 % of subjects affirmed to be truly interested in using the homeopathic approach for managing their cough (Fig. 7a and b).

**Fig. 6:**
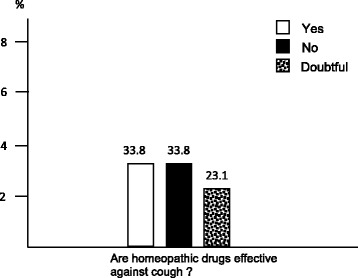
The subjects’ general opinion on homeopathic drugs

**Fig. 7:**
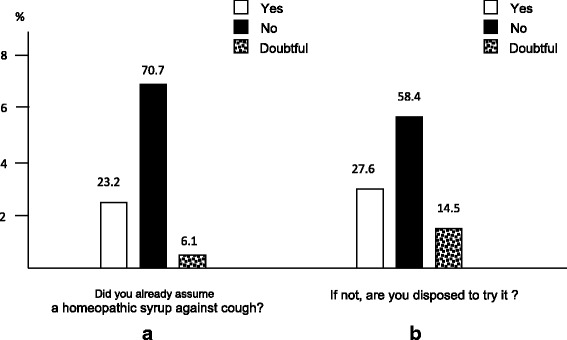
The subjects’ experience with (panel **a**) and disposition to (panel **b**) the use homeopathic drugs against cough
5. Willingness to pay
  Finally, the willingness to pay for an effective anti-tussive remedy was: 46.3 % of respondents accepted to pay up to 10 €; 27.8 % up to 20 €, and 13.3 % more than 20 € (Fig. 8).

**Fig. 8:**
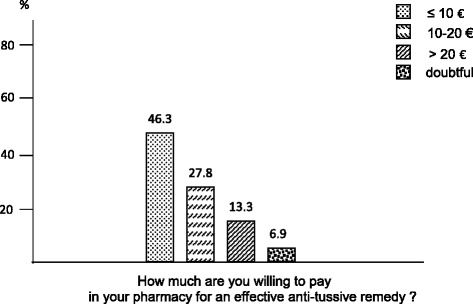
The patients’ willingness to pay for an effective anti-tussive drug

## Discussion

Despite cough is one of the most frequent events reported in respiratory medicine, its assessment still is not fully defined also from the scientific point of view in several conditions.

Actually, people’s perception not always corresponds to the etiological origin or the severity of cough, and subjects tend usually to be equally worried about their cough, particularly when long lasting, even in the absence of disabling conditions.

Unfortunately, there are some factors and variable conditions which can contribute to complicate the etiological definition, the discrimination of different clinical manifestations, and the management of cough in real life [1–3]. Also due to the poor subjects’ knowledge of cough, its perception can consequently result variable either by cougher and non-cougher individuals.

Even if some studies were carried out with the aim to investigate the prevalence of cough in our country, the perception of cough was not as well investigated among the general population in terms of people’s specific perception and beliefs; of their behavioural approach, and of therapeutic expectations.

Surprisingly, besides the consolidated subjects’ convincement that cough merely represents a non-specific symptom shared by several different diseases, it is quite interesting to pinpoint that, even if obviously fully unaware of the underlying scientific background, more than 46 % of respondents in the present survey supported the hypothesis that cough may represent a disease *per se*. Their position is particularly intriguing and challenging as it is fitting with the most recent scientific vision on cough, which actually regards cough as the transversal clinical effect of the “cough hypersensitivity syndrome”. In other words, cough is now suggested as a disease due to the hypersensitive condition of airway sensory nerves, independently of the particular etiology of cough [7–9]. In our opinion, this unexpected common vision between the general population and the scientific community will allow a new gate for a more effective entrance into the cough world, thus leading to ever interesting scientific developments in the next future.

Cough once again confirmed as a very common disorder occurring in general population, either in terms of incidence of episodes and of their duration. No particular behavioural tendency emerged from the present survey as the respondents’ declared actions for facing cough were equally ranging from “I do wait” to “I ask my Doctor for any aid”, mainly after 1 week of cough. This time interval appeared as the most frequent threshold since when the majority of people start to worry about their cough and to fear any possible respiratory complication, particularly if cough affects a child. In general, respondents proved well oriented on the possible causes of their cough as well as on the kind of specialist to refer to in case of clinical progression of the events.

Another interesting information came out from the analysis of the people’s beliefs on the therapeutic options to take against cough, and in particular against persistent cough. Actually, the great majority of respondents claimed to be not in favour of the immediate use of antibiotics and/or systemic steroids for managing cough. Once again, general population shared the right position of the scientific community, even if the prescription of these therapeutic options still is unfortunately extremely diffuse in clinical practice and attains also to a great proportion of GPs and lung physicians everywhere. In other words, people’s and doctors’ positions seem conflicting, even if it is well known that the consumption of antibiotics and/or systemic steroids, but also the self-medication with these drugs, are quite high in our country.

Furthermore, the great majority of respondents documented a strong attitude in favour of present anti-tussive drugs, and regarded the aerosols in general as the right therapeutic option against cough. From a generaI point of view, it should be pointed out that an effective treatment against cough is still missing in many cases, and many of the most prescribed products lack of strong evidence of efficacy. In particular, among over-the-counter (OTC) medications, symptomatic drugs (anti-tussives and mucolytics) are the most used, both as self-medication and as prescribed treatment after a medical consult [5]. Those preparations showed controversial results in clinical settings [10] and their use is not fully supported by guidelines, which, on the contrary, suggest to refrain from treatment in acute uncomplicated cough [10–13].

Besides the symptomatic treatment, there is still a high prescription of antibiotics [14, 15], although the evidence against their use is strong [16–18]. If the easy accessibility explains the wide use of the OTC products, several factors influence the high prescribing rates of antibiotics for acute uncomplicated cough, namely the doctor’s diagnostic uncertainty and the patient’s expectations [19]. Moreover, patient’s beliefs and expectations can be influenced by the high availability of uncontrolled health-related information [20, 21] which alter the delicate balance of the doctor-to-patient relationship [22, 23].

In terms of the therapeutic approach against cough, to note that more than one third of respondents affirmed their interest in homeopathic drugs. This is a substantial proportion, in our opinion, which tends to further confirm the general need of anti-tussive medications. This position is reinforced by the evidence that one out of four of respondents has already assumed homeopathic drugs (namely, an anti-tussive homeopathic syrup) for managing his cough, but also by the evidence that an ever higher proportion of respondents (such as, one third) claim their propensity to use a homeopathic remedy against cough.

Even if the present survey was not planned to investigate the etiology of cough and no data are available concerning the reasons of people’s attitude to anti-tussive therapeutic strategies, it is anyhow plausible that the high number of individuals that manifested such an increasing interest in homeopathic anti-tussive remedies would have likely be leaded to this disposition by the poor results obtained by using the traditional anti-tussive drugs presently available in the market. On the other hand, this hypothesis may be also supported by the increasing number of controlled trials aimed to investigate and compare the clinical effects of homeopathic versus usual anti-tussive drugs in recent years [24, 25].

Finally, the non complete satisfaction of public opinion concerning the efficacy of the present approach to cough treatment is clearly mirrored by their high disposition to pay a sum ranging from 10 to more than 20 € out-of-pocket for obtaining any effective anti-tussive remedy in pharmacy.

## Conclusions

Cough confirms its high impact in Italy, and at present a substantial proportion of individuals regard cough as “a disease”. Only one out of three adult Italians refers to his doctor, but when cough is already persistent. Cough in children is much more feared than in adults. The majority of Italians have a proper and restrictive position versus the use of both antibiotics and the steroids for treating cough. The Italian attitude to aerosol therapy confirms very high. Differently from the content of the current cough guidelines, anti-tussive drugs are highly valued among Italian people. Moreover, the attitude to homeopathic anti-tussive remedies proves high and still increasing. Finally, the willingness to pay for an effective anti-tussive remedy is quite high in Italy.

## Abbreviations

CATI, computer assisted telephone interview; URTIs, upper respiratory tract infections

## Additional file

Additional file 1: The questionnaire used for the interviews. (DOCX 19 kb)
