# Supplementary material for: Cough: impact, beliefs, and expectations from a national survey
Source: Multidiscip Respir Med. 2016 Sep 27;11:34. doi: 10.1186/s40248-016-0072-1 (PMC5037648; doi:10.1186/s40248-016-0072-1)
Supplement: Additional file 1: — The questionnaire used for the interviews. (DOCX 19 kb) [file 40248_2016_72_MOESM1_ESM.docx]

Additional file 1

**Introduction**

Hi, how are you ? We are carring out a National survey, and your opinion is quite important. May I put some simple questions concerning your believes on cough ? I’ll take maximum 5 minute of your time. The interview will remain anonymous and data collection is conducted according to the present law on privacy. If you agree, we can start (If Yes, go on; if not, tankyou, and have a good day).

**Questions**

1. **In your opinion, is cough a disease ?**

□ yes □ no □ doubtful

1. **What do you do after 2-3 days of cough ?**

1□ wait □ domestic remedies 3□ ask the Pharmacist 4□ ask the Doctor

1. **After when do you start to be worried of your cough ?**

□ 7 □ 15 □ 30

1. **Is cough merely a symptom of any disease ?**

□ yes □ no □ doubtful

1. **After 30 days of cough, what do you think about its cause ?**

…………………………………………

1. **Which specialist do you presume will be the best to refer to ?**

…………………………………………

1. **Should persistent cough only be effectively treated with antibiotics ?**

□ yes □ no □ doubtful

1. **Are present anti-tussive drugs effective ?**

□ yes □ no □ doubtful

1. **Are systemic steroids needed in persistent cough ?**

□ yes □ no □ doubtful

1. **Are domestic aerosols the right option against cough ?**

□ yes □ no doubtful

1. **Are homeopathic drugs effective against cough ?**

□ yesI □ no □ doubtful

1. **Did you already assume a homeopathic syrup against cough ?**

□ yes □ no □ doubtful

1. **If not, are you disposed to try it ?**

□ yes □ no □ doubtful

1. **How much are you worried if cough affects a child ?**

□ not at all □ as in adults □ more than in adults □ much more than in adults

1. **How much are you willing to pay in your pharmacy for an effective anti-tussive drug ?**

□ up to 10€ □ 10 - 20€ 3□ more than 20 €

1. **How many episodes of cough do you suffer over twelve months ?**

□ never □ 1-2 □ 3-5 □ more than 5

**( If “never”, skip to question # 20)**

1. **Which is the overall duration of these episodes ?**

□ never □ less than 10 □ 10-30 □ more than 30

1. **In general, does your cough produce any sputum ?**

□ yes □ no □ doubtful

1. **Age:** _______ (years)
2. **Gender:** □ male □ female
3. **Smoke:**  □ active □ never □ ex smoler
4. **Job:**

□ worker □ employee □⁮ manager □⁮professional/entrepreneur

□⁮ student □⁮ retired □⁮ unemployed □⁮ housewife

1. **Region where you live:** …………………………………..
